# Aphorisms, Etc.

**Published:** 1883-04

**Authors:** O. Buchmann

**Affiliations:** Alvensleben


### APHORISMS ON THE AFFINITIES OF MATTER IN ITS CONDENSATION AND ATTENUATION.

Dr. O. Buchmann in Alvensleben.

*From the Allg. Hom. Zeitung, Leipzig, 1883, Band 106, Nos. 3 and 4.*

In science only that is valued as property which is taught in the universities or transmitted by the past, and if anybody comes with something new which contradicts or threatens to overthrow the belief which we respected for years and communicated to others, all the passions rise up against him and all efforts are made to annihilate him. They resist with all their might, and act as if they could neither hear nor comprehend; they talk about the new view as if it were not worth the trouble of an investigation or even attention; and thus a new truth may wait a long time until it can make itself available. The greatest enemies of science are its specialists. In the profession is contained the guild with everything vulgar, raw, and egotistical of the trade.—*Goethe to Eckermann.*

The experiments of Crookes have shown that the molecules of the atmospheric air at a millionesimal attenuation are drawn asunder so far that they can unfold potentiated physical actions by freer motion in their ether.

The original state of aggregation of matter represented a much higher attenuation.

In order to show the gradual condensation, a short reference to the latest result of cosmological research from *Moldenhauer's Das Weltall und seine Entwickelung Koeln*, 1882, vol. II, may be given.

"We must descend to the far past, in order to observe step by step the development of our solar system. The earth does not yet exist at that time, nor any other planet. The sun of to-day is not yet in existence, and the elements constituting these worlds formed a ball in which there could not be any question of palpability, since the mass was composed of matter which was still a billion times thinner than our hydrogen gas. This is, so far as our power of comprehension is concerned, no more anything, no chaos, it is the 'NIHIL.' The so to say unsubstantial, shadowy ball, the primeval ball of our sun, of our planets and moons, has nevertheless already a past behind it. As formed cosmical individual, as ball, it has already lived through a period of development, and certainly not a short one. It is no more what it had been, no more the former mass of gas of a radius of about two billions of miles."

The ball, formerly six hundred thousand billion times thinner than hydrogen gas, has been contracted within itself. The condensation is a continuance of the act of conglobation in consequence of the attraction of the total mass upon the single particles, whereby a gravitation (falling) of the same toward the centre of the mass follows, and the gravitating effort of the particles in the outer sphere increases directly as the square with the shortening of the radius of the ball. The condensation reaches its end as soon as the particles of matter can approach no more.

By the rotation of the ball, which increases with the condensation, a gaseous ring from time to time has detached itself at the equator of the ball by the preponderance of the centrifugal force, which increases directly as the square of the velocity of rotation. From this ring, on bursting asunder and forming separate balls, the planets, and further on by detaching of rings from these, the moons have been formed, as the ring of Saturn teaches us, which has resisted the separation. At the time that the ring of the earth-planet had separated from the sun, this body had, with a radius of about twenty millions of miles, only a density of one nine-hundredth part of hydrogen gas, and the earth weighed at the separation of the moon with a radius of fifty thousand miles—still five and a half times less than an equal ball of hydrogen gas, which is fourteen times lighter than atmospheric air.

In comparison with the attenuation of substances in our high potencies, the primeval balls of the sun and of the planets appear still enormously dense.

The high temperature of the heavenly bodies caused by the continuous condensation of their particles, as the sun is showing still at the present time on his surface, was of great importance for the constitution of its mass.

Just as at that time the heat escaped in the macrocosmus, it now in the microcosmus becomes latent again in our attenuations.

At any rate, only with the decrease of the development of heat, the outer sphere of the earth was so cooled off that by aggregation of the like molecules a chemical association of the vapors became possible. The lighter vapors of the aluminium, silicium, magnesium, calcium, potassium, natrium, etc., combined with the oxygen at the surface to a fusible crust, while the vapors of the heavy metals, which in the interior of the earth had been overheated by the high gravitating pressure, could not, on account of the restlessness of the molecules, attain the condition of entering chemical composition. Thus, *e. g.*, one atom of silicium combined with two atoms of oxygen and formed a molecule of quartz, and two atoms of aluminium combined with three atoms of oxygen and formed alumina. Three parts of the first added to one part of the latter resulted in the formation of silicate of alumina. This, again, with another double compound of the silicate of potassium, has given rise to the crystalline orthoclase (feldspar), a main constituent of granite and other rocks.

In the further cooling of the earth's crust, we see arise the water as powerful solvent favoring new combinations. To the present day the silicic acid, which has been dissolved in quantities by the hot water, is again eliminated at the geysers near the Yellowstone Lake and at the Tetterata Spring.

Just as the combination of carbon, with the elements of the water, has increased its solving capacity by its amount of carbonic acid, so also the addition of an organic compound of carbon with the elements of water (alcohol) to water, increases the solving capacity of the same in the homœopathic attenuations. Substances difficult of solution become easier soluble by fine distribution (trituration).

Iridium, as found in small, white, metallic granules, is insoluble in all the acids, even in aqua regia, which dissolves platina and gold, but in finely divided condition it dissolves easier in aqua regia.

The chemical force of atoms is dependent upon their aggregation to molecules, which are so disposed as to approximate each other closely, and requires the at least partly liquid or gaseous state of aggregation, and sometimes the co-operation of other forces, in order to allow new groupings of atoms forming molecules of other composition.

Our chemists are far from believing the traditional atoms to be veritable "atomoi" (indivisibles), and hope, in the future, to be able to decompose some of them, *e. g.*, Chlorine. Of course, the chemical action as element which it was hitherto and is no more, would henceforth be suspended.

The molecules of the medicines have a partly nutritive, partly pathogenetic bio-chemical affinity to certain living cells.

The latter affinity, as a general rule, is stronger than the similar affinity of certain cells to certain natural causes of disease.

Inasmuch as the ingested molecules of the medicine come by the motion of the humors in contact with those cells which correspond to their affinity, the affinity to certain causes of disease is suspended by their stronger affinity.

If the remedy is adequate in form and dose, then the medicinal disease remains mostly latent, and the medicinal molecules are eliminated again by the restitutive power of the cells after the affinity is, as it were, *tired out.*

The physical properties of the substances have caused the physicists to assume the existence of infinitesimal, imponderable, ethereal atoms, which surround the molecules and which penetrate all substances, and thereby are enabled to mediate a physical action at a distance from the place of their origin.

Certain physiological experiments have furnished the proof that the ether-atoms of certain substances can exert a pathogenetic action *per distans.*

A similar penetration of very solid bodies with especial specific affinity already is shown by the molecules of the hydrogen gas. They penetrate platina at a red heat (also animal membranes which other gases cannot penetrate on account of the greater rise of their molecules.)

As the ether molecules of certain substances have a physical affinity to certain protoplasma molecules, so also the molecules of hydrogen gas have a physical affinity to the molecules of palladium.

If a rod of this metal is used as negative electrode of a water-decomposing apparatus, it is enabled by means of the electric current to absorb nine hundred times its own volume of hydrogen, whereby the volume of the palladium is increased and the specific gravity diminished. By heat the hydrogen molecules are again expelled and the palladium molecules are again condensated.

Some substances which readily emanate ether show the simultaneous action of ether already in the dense state. The emanation of ether is observed with some substances by the smell.

With metals the specific ether-smell develops by friction, *e. g.*, on grinding a knife.

The emanation of the ether molecules increases with the attenuation, while the chemical affinities decrease with the distance of the molecules from each other.

The action of ether, which appears only in the higher potencies, in the complex of symptoms which it is able to excite and to give by physical affinity, is different from the *bio-chemical* action of the unattenuated substance and of its lower potencies.

The low potencies of homœopathic medicines are apt to act more favorably where pathogenetic *bio-chemical* combinations with disease-substances exist, than the higher ones where *physical* pathogenetic combinations are present.

The experience teaches, that the disease-substances which thereby have been dissolved sometimes find still other affinities to metastases, even on the external skin, and at times at their elimination from the organism communicate specific quality and specific smell to the excretions, according to their chemical and physical influence.

Where experience does not speak for the selection of the potency, it will be advisable to give the simillimum first in low potency, and when this does not suffice to apply the higher one.

The physical ether-combination with some remedy appears accompanied by considerable disturbances of the function, while the medicine-disease caused by *bio-chemical* combination with the same remedy frequently in lower attenuation remains latent.

There are persons, especially incurable patients, who by taking high potencies, especially after repetition, feel so sick that they cannot be induced to take them again.

There are persons who cannot bear high potencies, and yet are not affected materially by strong purgatives and narcotics, though the reverse also is frequently observed.

Some persons do, as a general rule, not bear certain remedies at all.

There are persons who recognize the high potencies of certain remedies by smell, taste, and other sensations as the remedies which they had taken previously, even a long time before.

The power which ether-atoms at greater density, therefore, not too far from their original place, are able to exert *per distans* where they meet molecules, to which they are attracted by a specific affinity, is shown very distinctly by the affinity of magnetism for iron.

The magnetic ether penetrates everything in order to reach the iron molecules, and the attractive force is so great that the gravitation of iron is thereby overcome.

The magnetic imponderable ether-atoms are polarly fixed in a steel rod by the aid of motion, and can be transmitted infinitesimally from one steel rod to others without ever exhausting the magnetic force.

A similar transmission of the ether atoms of our high potencies at every centesimal attenuation of the same *ad infinitum* by the aid of motion (succussion) follows without exhausting the pathogenetic force.

The action of magnetism upon iron is a greater enigma than the action of high potencies. The first, though universally used, is only stared at by the physicists as incomprehensible, while the latter, as mystical, is by the learned tribe thought unworthy of investigation.

Similar to the mineral magnetism, the ether-atoms of the animal magnetism also have a specific affinity to the ganglionic cells, to the acknowledgment of which the learned crowd has been forced only in the latest time.

There must be a special individual disposition in order to make the physical affinity of certain cells to certain ether-atoms recognizable as pathogenetic combination, a reason why not everybody is fit to be a prover.

Hence the objection, that because pathogenetic and physiological actions cannot be observed upon everybody, those observed upon single individuals should not be considered as of scientific value, is entirely futile.

The physical combination of certain cell-molecules with the ether-atoms of a substance is able to diminish and suspend the *bio-chemical* affinity to the molecules of the same substance, and therefore it is explainable that the high potency of a remedy acts as the antidote to excessive doses and symptoms of intoxication by the same medicine, and that the homœopathic attenuation of a disease-substance can become the remedy for the *bio-chemical* combination of this disease-substance. (Isopathy. Vaccination.)

By pathogenetic combinations of certain cells with certain causes of disease, the electro-nutritive affinity of the same is disturbed, so that certain nutritive substances cannot properly be suscepted and separated, which then causes disturbances of function.

The cells which have a *bio-chemical* nutritive affinity to certain mineral substances possess also a greater *physical* affinity to the ether-atoms of the same substances than to the cause of the disease which has disturbed the *nutritive* affinity of these cells.

Hence, by introducing the ether-atoms of these substances, the pathogenetic affinity to the cause of the disease is suspended, and the *bio-chemical* nutritive affinity restored.

It is Schuessler's merit, also without previous physiological proving of their affinities as homœopathically acting remedies, to have given the indications of several anorgano-chemical combinations by considering the nutritive affinity.

The *bio-chemical* affinity of these nutritive substances cannot be active in a cure by the same, since their *bio-chemical* affinity is weakened by the stronger affinity to the cause of the disease. Hence higher potencies of the remedies of Schuessler will accomplish more than less triturated larger doses in order to gain a stronger physical action.

It is an irrational procedure to try to replace anorganic nutritive substances by greater ingestion of the same, since they are already amply existing in the common food in a more assimilable form.

